# The Ability of Acid-Based Natural Deep Eutectic Solvents to Co-Extract Elements from the Roots of *Glycyrrhiza glabra* L. and Associated Health Risks

**DOI:** 10.3390/molecules27227690

**Published:** 2022-11-09

**Authors:** Alexander N. Shikov, Veronika A. Shikova, Anastasiia O. Whaley, Marina A. Burakova, Elena V. Flisyuk, Andrei K. Whaley, Inna I. Terninko, Yulia E. Generalova, Irina V. Gravel, Olga N. Pozharitskaya

**Affiliations:** 1 Department of Pharmaceutical Formulations, St. Petersburg State Chemical Pharmaceutical University, 14 Prof. Popov, 197376 Saint-Petersburg, Russia; 2 Department of Industrial Technology of Medicines, St. Petersburg State Chemical Pharmaceutical University, 14 Prof. Popov, 197376 Saint-Petersburg, Russia; 3 Department of Pharmacognosy, St. Petersburg State Chemical Pharmaceutical University, 14 Prof. Popov, 197376 Saint-Petersburg, Russia; 4 Core Shared Research Facilities “Analytical Center”, St. Petersburg State Chemical Pharmaceutical University, 14 Prof. Popov, 197376 Saint-Petersburg, Russia; 5 Department of Pharmaceutical Natural Science, Institute of Pharmacy Named after A.P. Nelyubin, I.M. Sechenov First Moscow State Medical University (Sechenov University), 8/2 Trubetskaya, 119991 Moscow, Russia; 6 Murmansk Marine Biological Institute of the Russian Academy of Sciences (MMBI RAS), 17 Vladimirskaya, 183010 Murmansk, Russia

**Keywords:** extraction, licorice, *Glycyrrhiza*, metal pollution, NADES, trace elements, jlycyrrhizic acid

## Abstract

The roots of licorice (*Glycyrrhiza glabra* L.) have been widely used in traditional and officinal medicines for the treatment of different diseases. Natural deep eutectic solvents (NADES) have become popular for the extraction of active principles from medicinal plants. However, the ability of NADES to co-extract trace elements during the isolation of target active compounds is rarely investigated. The aim of this study was to analyze the content of trace elements in acid-based NADES extracts from the roots of *G. glabra* and the health risks associated with them. In this study, we have tested for the first time the ability of several acid-based NADES to co-extract glycyrrhizic acid (GA) and trace elements from the roots of *G. glabra*. GA has been identified as the dominant phytochemical in *G. glabra* NADES extracts (0.145–0.495 mg/g). Due to the close pKa of lactic acid and GA, the yield of GA in lactic acid-based NADES was higher in comparison with other tested NADES. The yield of GA in NADES3-NADES5 was statistically significant and surpassed the yield of GA in water. The recovery of all elements (except Li) by all tested NADES was low (less than 6%). According to an ANOVA test, the hydrogen bond donor type plays a decisive role in the extraction of elements. A strong positive correlation between the recovery of GA and MPI was noted. The metal pollution index, hazard quotient, hazard index, and chronic daily intake were calculated and suggest that all tested NADES extracts of *G. glabra* roots were nontoxic and possess no health risk for both ingestion and topical application.

## 1. Introduction

Licorice, *Glycyrrhiza glabra* L. (*Fabaceae*), is a well-known medicinal plant in both European and Asian medicine. This perennial plant is widely distributed throughout Russia including the European part, South, and West Siberia [1]. The roots of *G. glabra* together with the roots of *G. uralensis* Fisch. are used in Russian official medicine and monographed in the State Pharmacopoeia of the Russian Federation as Glycyrrhizae radices [2]. The medical use of licorice is approved by the European Medicines Agency [3,4]. In traditional Chinese medicine, licorice is regarded as a “guide drug” and added to polyherbal compositions to enhance the bioavailability, reduce toxicity, and disguise the taste of other ingredients [5]. Licorice is used as a therapy for fatigue, exhaustion, catarrhs, upper respiratory tract and viral diseases, asthma with coughing, and as an expectorant [2,4,6,7,8]. More than 400 biologically active compounds have been identified in licorice, among which triterpenoid saponins (glycyrrhetinic acid) and flavonoid compounds dominate [8,9].

Starting with the pioneering work by Prof Verpoorte’s group [10], natural deep eutectic solvents (NADES) are the focus of scientists as green, ecologically friendly, and non-toxic solvents for the extraction of different groups of biologically active compounds from natural sources [11,12,13,14,15]. A key advantage of NADES as perspective solvents is that they can be tuned to have different functionality owing to diverse possible combinations of starting components [11,14,16]. This vital feature permits NADES to explore newer opportunities in the field of natural ingredient extraction. Recently, NADES has been reported for the extraction of glycyrrhizic acid [17], licuroside [18], glabridin, and isoliquiritigenin [19] from *G. glabra*. Due to an increased industrial load on the environment, medicinal plants could accumulate a high amount of trace elements [20,21] which are transferred with solvents from the plant material to the extract. The trace elements can be recovered from plant material by NADES [22,23]. However, the ability of NADES to co-extract trace elements from the roots of *G. glabra* has not yet been investigated.

This study is aimed at the analysis of trace element content in acid-based NADES extracts from the roots of *G. glabra* and the potential associated health risk.

## 2. Results and Discussion

The majority of commonly used NADES are polar [24]. Acid-based NADES were reported as effective solvents for the extraction of different compounds from licorice [17,18,19,25]. Based on literature data and our previous experiments, we have used the following NADES in our experiments (Table 1).

### 2.1. Content of Glycyrrhizic Acid in NADES Extracts

Glycyrrhizic acid (GA) is one of the key active principles in licorice. Five different NADES (Table 1) were prepared to assess their extraction efficiencies for GA. Here, sucrose, sorbitol, and choline chloride were used as hydrogen bond acceptors (HBA), and two carboxylic acids such as citric acid and lactic acid as hydrogen bond donors (HBD).

**Figure 1:**
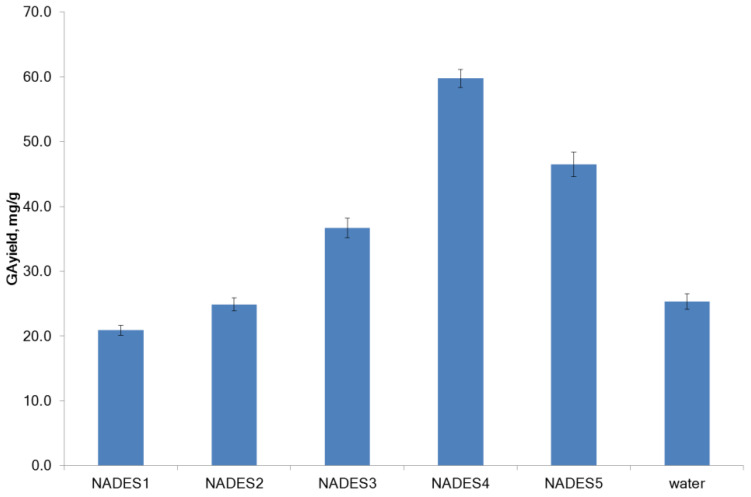
The yield of glycyrrhizic acid in NADES and water.

The concentration of GA was analyzed by high-performance liquid chromatography (HPLC). HPLC is a useful method for the analysis of plant extracts simultaneously tested and analytically compared without time-consuming fractionation [26]. The typical chromatogram of NADES extract of licorice, the chromatogram of GA, and NADES are presented in Figure S1. GA dominates in all NADES extracts, while its concentration in extracts varied from 0.145 to 0.495 mg/g. The yield of GA in the citric acid-based NADES was equal to the yield of GA in water, while the efficacy of lactic acid-based NADES was statistically and significantly higher compared with citric acid-based NADES (Figure 1).

Our results are in agreement with those previously published. Lanjekar et al. [25] have reported that the NADES based on organic acids (citric, lactic, malic, oxalic, and succinic) were more effective for the extraction of GA from licorice roots than the sugar- and polyalcohol-based NADES. When discussing the differences observed in the extraction efficiencies, the NADES polarity should be considered. Being a moderately polar compound, GA is more soluble in polar solvents than in non-polar solvents. The high yield of GA was obtained in lactic acid-based NADES. The lactic acid-based NADES possesses pH (~1.7), which is close to the pKa value of GA, which could be one of the reasons behind the high extraction yield.

### 2.2. Co-Extraction of Trace Elements with NADES

Many trace elements are represented in medicinal plants in the form of mineral salts. Being polar, they are capable of co-extraction with NADES. To understand the ability of elements for co-extraction with the target active compound (GA) from licorice roots, we have analyzed the content of several elements in licorice root and NADES extracts from *G. glabra*.

The total content of elements in the dry roots of *G. glabra*, which was determined by inductively coupled plasma atomic emission spectroscopy (ICP-OES), was 19,095 ± 324 mg/kg DW (RSD 1.70%). The concentration of individual elements was: Al—132 ± 3 mg/kg DW (RSD 1.67%); Ca—12,570 ± 364 mg/kg DW (RSD 2.89%); Cu—3.67 ± 0.04 mg/kg DW (RSD 1.09%); Fe—146 ± 2 mg/kg DW (RSD 1.02%); K—2470 ± 64 mg/kg DW (RSD 2.59%); Li—0.120 ± 0.002 mg/kg DW (RSD 1.67%); Mg—2552 ± 63 mg/kg DW (RSD 2.47%); Mn—13.3 ± 0.2 mg/kg DW (RSD 1.28%); and Na—1208 ± 40 mg/kg DW (RSD 3.31%). Licorice roots contain relatively high amounts of Ca, Mg, K, and Na, while the concentrations of Al, Fe, and Mn were relatively lower. The lowest concentrations among the analyzed elements were obtained for Cu and Li.

The information about element composition in licorice roots is rarely reported. The difference in element content in licorice roots depends on the collection site and other factors. The average K, Ca, Mg, Fe, Mn, Na, and Cu composition measured using an atomic absorbance method in *G. glabra* roots from the Eastern Anatolia region of Turkey was 14,000 mg/kg, 1470 mg/kg, 1200 mg/kg, 200 mg/kg, 60 mg/kg, 21 mg/kg, and 1 mg/kg, respectively [27]. The synchrotron X-ray fluorescence method was used for elemental analysis of Glycyrrhiza uralensis growing in natural habitats of Russia (Novosibirsk region, Altai Territory, Republics of Khakassia, Tuva, and Buryatia), East Kazakhstan, and Kyrgyzstan. It was established that the underground parts of licorice accumulate significant concentrations of Mn (3.8–274 mg/kg), Fe (65–4531 mg/kg), and Cu (3.2–27 mg/kg) [28]. The results are consistent with earlier studies [27,28].

**Figure 2:**
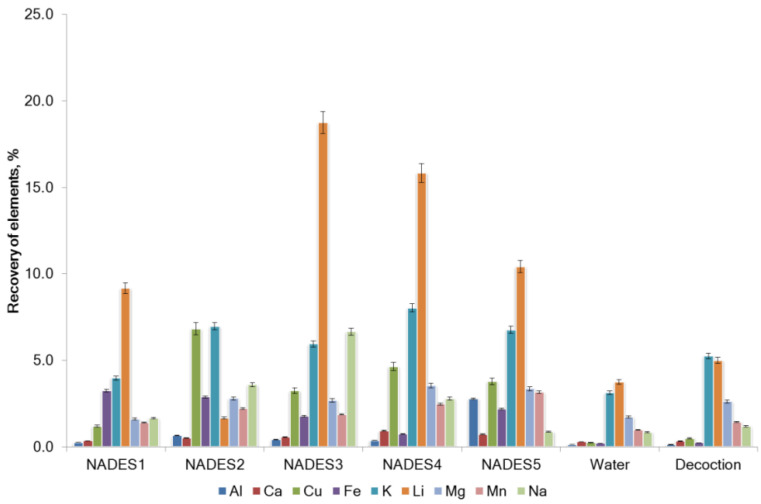
Recovery of the elements from *G. glabra* using NADES and water.

**Figure 3:**
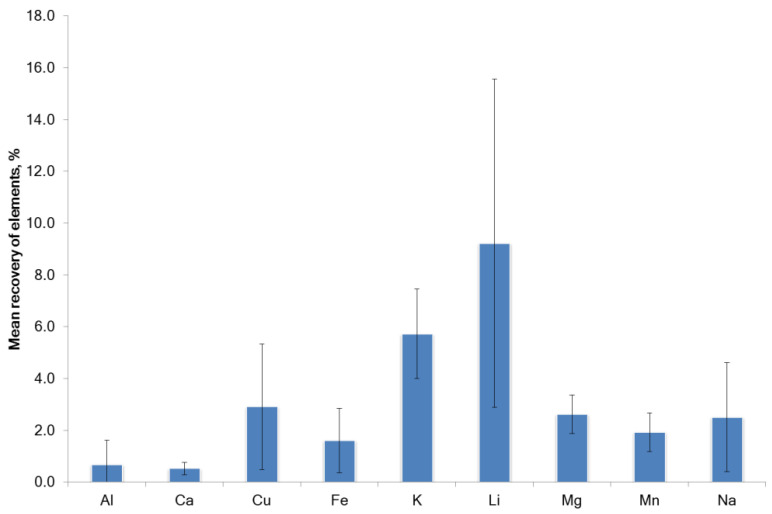
Mean recovery of the elements from *G. glabra* using NADES and water.

The roots of *G. glabra* were extracted as described in Section 2.3. Significant differences in the co-extraction of trace elements with the tested NADES were observed in the concentration profiles (Table 2). The calculated element recovery is shown in Figure 2 and Figure 3. Low recovery (<6% recovery) for all elements tested except for Li was found in the NADES extracts (Figure 3). Recovery of Li was relatively high and varied considerably (from 1.67% to 18.75%). A direct correlation was found between the content of the element in the raw plant material and the concentration of the element in the extracts. In general, the ability of NADES3-NADES5 for the co-extraction of trace elements was greater in comparison with other solvents. The difference in the recovery of elements in the water extract and decoction was not statistically significant (*p* = 0.14).

Previously, it was shown that the transfer of individual elements from the spasmolytic herbal tea (herbal mixture) to the decoction ranged from 2.30% to 50.39%. At the same time, smaller recoveries were found for elements with high concentrations in the raw material (Fe), and the largest ones were found for elements whose level in the raw material was insignificant (Mo and Cd) [29]. It was found that the recovery of Mn in decoctions ranged from 2% to 29%, from 3% to 9% for Fe, from 2% to 20% for Cu, and from 14% to 50% for Mo. When testing 21 herbal teas, it was found that with an increase in the concentrations of trace elements in herbal teas, their concentrations in decoctions (water extracts) increase, and the recovery decreases and does not exceed in most cases for Fe—10%, Zn, Mn, and Cu—40%, and Co—20% [20]. In the study of the herbal tea used for the treatment of cough and cold (containing chamomile flowers, licorice roots, peppermint leaves, wild rosemary shoots, calendula flowers, and violet herb) it was found that the concentrations of macronutrients decreased in the series K > Ca > Mg > P > S > Na > Fe, while the recovery was 10%–80% [30]. In the current study, the same range of elements was observed among the analyzed elements in both NADES extracts and aqueous extracts.

**Figure 4:**
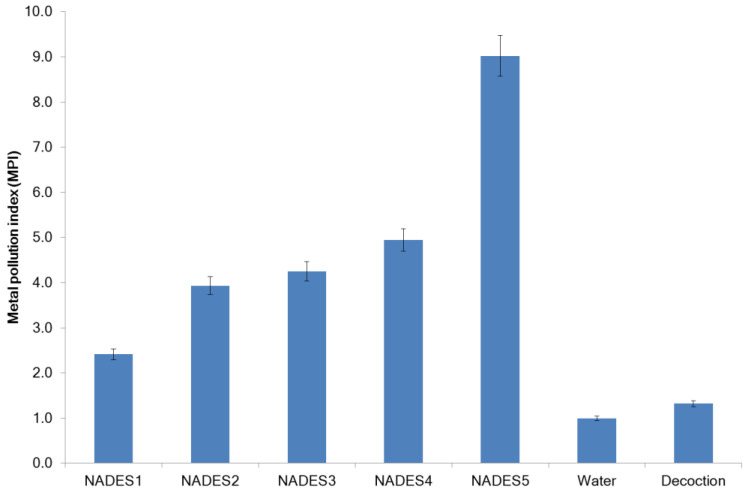
Cumulative accumulation of metals (MPI).

Metal pollution indices (MPI) for all studied extracts were calculated using the average concentrations of all studied elements (Figure 4). The MPI calculated using the average values of all samples of *G. glabra* NADES extracts turned out to be 4.9 ± 2.5 and varied from 2.4 (NADES1, sucrose-citric acid 3:1, 30% water) to 9.0 (NADES5, choline chloride—lactic acid 1:3, 30% water). A multivariate ANOVA test showed a significant effect on the overall co-extraction of MPI metals (*p*-value < 0.05) of both the hydrogen bond donor (the degree of influence of the factor is 58.9%, *p*-value 0.018) and hydrogen bond acceptor types (the degree of influence of the factor is 7.7%, *p*-value 0.033). The correlation between the concentration of GA content in NADES extracts and MPI was positive and strong (Pearson’s correlation coefficient r = 0.914, *p* < 0.05). The effect of the interaction between the composition of NADES does not affect the value of the MPI. The MPI for licorice root was 130.3 ± 2.4.

### 2.3. Human Health Risk Assessments of NADES Extracts

**Figure 5:**
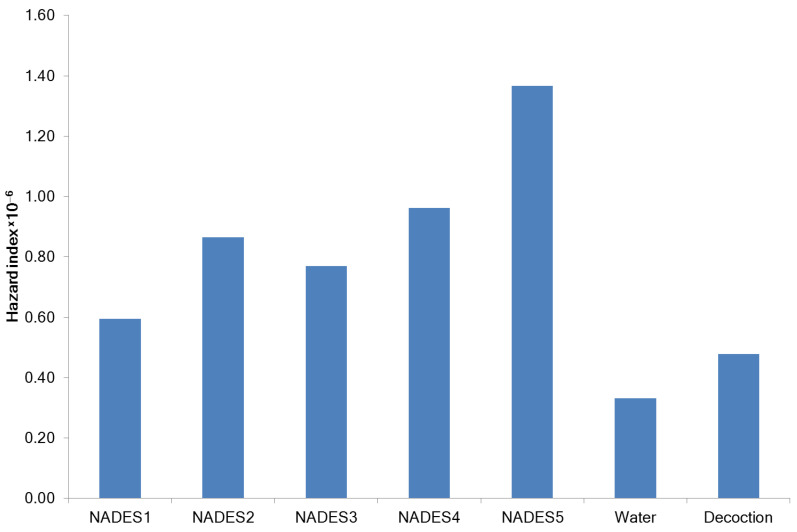
Non-carcinogenic risk (HI) of *G. glabra* extracts.

The daily intake dose of plant extracts contaminated with heavy metals determines the toxicity of the extracts [31]. The pollutants could enter the human body by inhalation through the mouth and nose, penetration through the skin, and oral administration. NADES have negligible vapor pressure [32]. Therefore, assessing the health risk of inhaling NADES extracts is not rational. NADES extracts are used for topical application. The hazard quotient (HQ) was used for the health risks assessment of tested extracts in the case of topical application. HQ is the ratio between the calculated dose of a pollutant and a reference dose level. HQ > 1 suggests a possible negative impact of the tested product [33]. The HQ levels for Al, Cu, Fe, Li, and Mn were calculated as 5.37 × 10^−9^, 2.93 × 10^−8^, 1.39 × 10^−8^, 2.25 × 10^−8^, and 8.40 × 10^−7^, respectively. The calculated HQ values for all tested metals were considerably less than 1, indicating that most likely no significant health risk exists after the topical application of the studied NADES extracts and aqueous extract. The combined non-carcinogenic effect of elements was expressed by the hazard index (HI). The mean HI for NADES extracts from *G. glabra* (HI = 9.11 × 10^−7^ ± 2.88 × 10^−7^), as well as from aqueous extracts and decoctions (HI = 4.04 × 10^−7^ ± 1.04 × 10^−7^), was less than 1 (Figure 5). This potentially suggests that no carcinogenic risk is associated with topical applications.

The USEPA model and its threshold values [1989] were used to assess the potential human health risks posed by heavy metal pollution [34,35]. The mean concentration of each element in NADES extracts was used for the calculation of the chronic daily intake (CDI) of all tested elements. The mean CDI of Al, Ca, Cu, Fe, K, Li, Mg, Mn, and Na was calculated as 5.4 × 10^−9^, 1.9 × 10^−7^, 1.2 × 10^−9^, 9.7 × 10^−9^, 3.9 × 10^−7^, 4.5 × 10^−11^, 1.7 × 10^−7^, 8.1 × 10^−10^, and 1.3 × 10^−7^ mg/day, respectively. The calculated daily consumption of all the metals with NADES extracts was less than the reference daily dose risk estimators (Table 3). In NADES extracts, average CDI values increased in the following order: Li < Mn < Cu < Al < Fe, Na < Mg < Ca < K.

The calculated amount of several elements in a daily dose of NADES extracts, water extract, and decoction of *G. glabra* let us understand the benefits/risks of a daily intake of licorice extracts. The dose of one tablespoon (20 g) of licorice NADES extracts was considered the average daily consumption. The maximal concentration of each element and relevant NADES extract are presented in Table 3. Then, the daily dose for each element consumed with 20 g of relevant NADES extracts from *G. glabra* was calculated and compared with the risk estimations for a 70-kg man [36,37,38,39] and nutritional requirements [39,40]. The data presented in Table 3 show that NADES extracts of *G. glabra* are non-toxic and could be considered a valuable source of dietary elements that meet daily nutritional requirements.

The possible co-extraction of trace elements from raw plant materials with NADES requires specific attention due to the eventual increase in the toxicity of extracts. However, the co-extraction of elements during the isolation of target compounds from raw plant materials is not well investigated. Tsvetov and Drogobuzhskaya [22] have reported relatively high concentrations of Si, Zn, Cu, Ni, and Mn in acid-based DES extracts from *Empetrum nigrum* L. In our recent report, acid-based NADES were shown to be able to co-extract several trace elements from *Fucus vesiculosus* [23]. The recovery of trace elements by NADES was less than 9%. The hazard quotient (HQ < 1) and hazard index (HI < 1) for all tested elements indicated no potential health risk associated with NADES extracts from *F. vesiculosus*. In the current study, acid-based NADES provide effective extraction of GA from licorice roots. Although the GA content was positively correlated with MPI, and the level of trace elements in NADES extraction was not high and did not pose a health risk.

## 3. Materials and Methods

### 3.1. Materials and Reagents

The roots of *G. glabra* L. were provided by Krasnogorskleksredstva JSC (Krasnogorsk, Russia). Choline chloride was purchased from Acros Organics (Fair Lawn, NJ, USA), L-lactic acid was from Panreac Química SLU (Barcelona, Spain), and sucrose, sorbitol, and citric acid were from Reachim JSC (Moscow, Russia). The glycyrrhizic acid (97.1% purity) was from Sigma-Aldrich RTC, Inc. (Laramie, WY, USA). Chromatographic HPLC acetonitrile was purchased from T. Backer (Gliwice, Poland).

### 3.2. NADES Preparation and Extraction Conditions

The NADES were prepared through the heating method [41]. Lactic and citric acids and hydrogen bond donors (sucrose, sorbitol, or choline chloride) at the respective molar ratio were used. The pre-weighed components were gently mixed and heated in a water bath at 50 °C for 60 min with agitation at 700 rpm, and 30% of water (*v*/*v*) was added to all NADES. Extraction (0.5 g of roots with 20 mL of NADES or water) was performed with stirring (600 rpm) at 50 °C for 30 min. Decoctions from licorice roots were prepared by heating 0.5 g of licorice roots with 20 mL of water for 30 min in the water bath.

### 3.3. Analysis of Glycyrrhizic Acid Content

The analysis was performed by HPLC [42] with some modifications. Briefly, 200 mg of NADES extracts were dissolved in 0.4 mL of 70% EtOH and injected into a Prominence LC-20 Shimadzu HPLC system (Shimadzu, Japan) equipped with a degasser (DGU-20A), column oven (CTO-20AC), a diode-array detector (SPD-M20A), autosampler (SIL-20A), and a low-pressure gradient pump (LC-20AD). A SUPELCOSIL LC18 column (25 cm × 4.6, 5 µm particle size) purchased from Merck was employed for the analysis (Darmstadt, Germany). The HPLC mobile phase was made up of low-pressure gradient elution system. The eluent was solvent A: H_2_O + 0.1% TFA and solvent B: CH_3_CN + 0.1% TFA. The following gradient elution was applied: 0–5 min, 10–50% B; 5–20 min, 50% B; 20–25 min, 50–10% B. The flow rate was set at 1 mL/min using 254 nm as the wavelength for UV detection. The column temperature was set at 40 °C with an injection volume of 10 µL.

The eluted GA was quantified using the following Equation (1) [25]:

(1) GAyield (mgg)=GAconcentration (mgL)×Vextractant(L)mlicorice(g)

The concentration of GA was calculated using a calibration curve. The calibration curve was linear at the concentrations range 0.01–0.5 mg/mL (R^2^ = 0.9999); LOD = 0.0040 mg/mL; LOQ = 0.0122 mg/mL. The typical chromatogram of NADES extract of licorice, the chromatogram of GA, and NADES are presented in Figure S1.

### 3.4. Elements Analysis

The concentration of metals such as aluminum (Al), calcium (Ca), copper (Cu), iron (Fe), potassium (K), lithium (Li), magnesium (Mg), manganese (Mn), and sodium (Na) was analyzed in triplicates using an Inductively Coupled Plasma Optical Emission Spectrometer (ICP-OES) Perkin Elmer Optima 8000 (Perkin Elmer Inc., Shelton, CT, USA). Microwave digestion unit Speedwave Entry Two (Berghof, Eningen unter Achalm, Germany) was used for the decomposition of plant and NADES samples before analyses by ICP-OES as described previously [23]. Analytical signals were measured as emission intensity values.

The total content of metals was obtained from three independent experiments, and the mean value was calculated for each metal. Relative standard deviations (RSD) among replicates of analysis of each sample were always lower than 4%. The R^2^ values ranged from 0.9990 to 0.9999, confirming the linearity of the calibration curves. The limits of detection (LOD) and quantification (LOQ) are defined as the concentration corresponding to three and six times the standard deviation (SD) of the blank, respectively, divided by the slope of the calibration curve. The LOD was calculated for standard deviations (SD) of six measurements for blank. The accuracy of the method was evaluated by adding a reference sample of Cu to a licorice sample, followed by extraction with NADES or a standard solvent. The recovery value of Cu was obtained from three independent experiments, and the mean value was between 89% and 105%. Hence, the results obtained from validation parameters confirmed that the applied method ICP-OES fulfills the required criteria set by the Association of Official Analytical Chemists [43].

The yield of extraction (recovery, %) was calculated as follows according to Equation (2):

(2) $$Recovery=\frac{M_{n}-M_{NADES}}{M_{licorice\;root}}\times 100$$

where *M*_n_**, *M_NADES_*, and *M_licorice root_*—concentration of the metal *n* in the sample, pure NADES and licorice root in mg/kg.

### 3.5. Metal Pollution Index

The metal pollution index (MPI) [23,44] is a mathematical model that summarizes the composite influence of all the elements in the extract and is calculated as the mean of values for the metals considered using Equation (3) as follows:

(3) $$MPI={\left(M_{1}\times M_{2}\times \ldots \times M_{n}\right)}^{1/n}$$

where *M_n_*—concentration of the metal *n* in the sample in mg/kg.

### 3.6. Human Health Risk Assessments

The nutrimental importance of essential elements was assessed on the basis of nutritional requirements [40]. The health risk due to the toxic elements present in seaweeds was estimated using risk estimators [36,37,39,40].

The chronic daily intake (*CDI*) was calculated by Equation (4), and the detailed explanation for all of the parameters is listed in [33]. Equation (4) is adapted from the USEPA [45].

(4) $$CDI=\frac{CS\times SA\times AF\times ABS\times EF\times ED\times CF}{BW\times AT}$$

where *CDI* is the chronic daily intake through dermal absorption in mg/kg/day; *CS* is the average concentration of metal in an extract of *G. glabra* in mg/kg; *SA* is the exposed skin area in cm^2^; *AF* is the adherence factor in cm^2^/mg; *ABS* is the dermal absorption fraction; *EF* is the exposure frequency in day/year; *ED* is the exposure duration in years; *CF* depicts the units conversion factor in kg/mg; *BW* is the average body weight in kg; and *AT* is the averaging time in days for non-carcinogens.

The human health risk assessment was performed by calculating the hazard quotients (*HQ*, non-carcinogenic risk from individual metals) for metals using Equation (5).

*HQ* = *CDI_dermal_*/*RfD* (5)

where *RfD* is the chronic reference dose of the toxicant in mg/kg/day.

People who consume herbal extracts are not only exposed to one metal but more than one metal in that extract. Thus, the hazard index (*HI*) was calculated by adding the *HQ* values of each metal in the study (Equation (6)). While *HI* < 1 indicates no concern about the potential health risk, *HI* ≥ 1 indicates a potential health hazard [46].

*HI* = ∑*HQ* (6)

### 3.7. Statistical Analysis

Data were subjected to statistical analysis using multi-factorial analysis of variance (ANOVA) to determine the effect of independent variables on metal co-extraction from *G. glabra*. All statistical calculations were performed with STATGRAPHICS Centurion XV (StatPoint Technologies Inc., Warrenton, VA, USA). Data are expressed in the form of the mean ± standard deviation (±SD), and error bars in the figures indicate the associated standard deviation.

## 4. Conclusions

Being polar solvents, acid-based NADES are able to co-extract trace elements from raw plant materials. In this study, we have tested for the first time the ability of several acid-based NADES (sucrose:citric acid 3:1, sorbitol:citric acid 3:1, sucrose:lactic acid 3:1, sorbitol:lactic acid 3:1, and choline chloride:lactic acid 1:3) to co-extract trace elements and GA from the roots of *G. glabra*. Due to similarities in the pKa of lactic acid and GA, the yield of GA in lactic acid-based NADES was higher in comparison with other tested NADES. All *G. glabra* root extracts were simultaneously tested and analytically compared without time-consuming fractionation. GA has been identified as the dominant phytochemical in *G. glabra* roots. The content of GA in NADES extracts of licorice varied from 0.145 to 0.495 mg/g. The yield of GA in NADES3-NADES5 was statistically significant and surpassed the yield of GA in water. The recovery of all elements (except Li) by all tested NADES was low (less than 6%). According to the ANOVA test, the hydrogen bond donor type plays a decisive role in the extraction of elements. A strong positive correlation between the recovery of GA and MPI was noted. Calculated metal pollution index, hazard quotient, hazard index, and chronic daily intake suggest that all tested NADES extracts of *G. glabra* roots were non-toxic and should possess no potential health risk after both topical application and ingestion.

## Figures and Tables

**Table 1:** NADES used for the extraction of licorice roots.

NADES Code	Composition	Ratio
NADES1	Sucrose:Citric acid	3:1
NADES2	Sorbitol:Citric acid	3:1
NADES3	Sucrose:Lactic acid	3:1
NADES4	Sorbitol:Lactic acid	3:1
NADES5	Choline chloride:Lactic acid	1:3

**Table 2:** The concentrations of elements (mg/kg) in samples of *Glycyrrhiza glabra* extracts (mean ± SD, n = 3).

Elements	Wave Lenght (nm)	LOQ (µg/L)	NADES1	NADES2	NADES3	NADES4	NADES5	Water	Decoction
Al	396.153	86	1.19 ± 0.05	2.25 ± 0.03	1.40 ± 0.02	1.61 ± 0.06	5.10 ± 0.05	0.183 ± 0.021	0.160 ± 0.011
Ca	317.933	198	47.5 ± 0.6	68.1 ± 0.3	76.5 ± 0.7	123 ± 54	97.1 ± 0.1	36.7 ± 0.4	41.0 ± 1.0
Cu	327.393	17	0.096 ± 0.017	0.309 ± 0.023	0.169 ± 0.012	0.204 ± 0.002	0.172 ± 0.011	0.060 ± 0.018	0.069 ± 0.006
Fe	238.204	12	5.43 ± 0.027	45.5 ± 0.1	3.94 ± 0.04	2.42 ± 0.07	4.96 ± 0.40	0.286 ± 0.032	0.332 ± 0.006
K	766.491	48	114 ± 2	175 ± 2	166 ± 1	203 ± 4	177 ± 2	78.0 ± 5.7	130 ± 2
Li	670.784	9	0.007 ± 0.001	0.012 ± 0.001	0.022 ± 0.001	0.025 ± 0.002	0.031 ± 0.002	0.002 ± 0.001	0.003 ± 0.001
Mg	279.077	47	41.4 ± 0.5	72.3 ± 0.1	69.2 ± 0.4	91.6 ± 2.2	87.2 ± 0.4	43.9 ± 5.9	66.8 ± 0.6
Mn	257.610	38	0.230 ± 0.001	0.333 ± 0.001	0.294 ± 0.001	0.373 ± 0.009	0.493 ± 0.004	0.133 ± 0.013	0.192 ± 0.002
Na	589.592	64	13.60 ± 0.01	21.9 ± 0.1	87.5 ± 0.1	105 ± 6	40.3 ± 0.6	12.1 ± 1.5	16.2 ± 0.4

**Table 3:** Element maximum concentration (mg/kg), its daily dose (mg/day) in the extracts from *G. glabra*, and comparison with daily dose risk estimators for a 70-kg man and nutritional requirements.

Element	NADES	Maximum Concentration	Daily Dose for 20 g Consumption	Daily Dose from Risk Estimators	Daily Nutritional Requirements
Al	NADES5	5.1	0.102	10 ^1^	
Ca	NADES4	123.1	2.462	2500 ^2^	1000 ^3^
Cu	NADES2	0.31	0.006	5 ^2,5^	0.9 ^4^/1.0 ^5^
Fe	NADES1	5.43	0.109	45 ^5^	10 ^3,5^
K	NADES4	203.35	4.08	3500	2500
Li	NADES5	0.031	0.0006	0.3	0.1
Mg	NADES4	91.61	1.83	800 ^5^	400 ^5^
Mn	NADES5	0.49	0.01	11 ^5^	2.7 ^3^/2.0 ^5^
Na	NADES4	104.80	2.10	2000 ^2^	

^1^ PTWI: Provisional tolerable weekly intake; ^2^ UL: Tolerable upper intake level; ^3^ PRI: Population reference intake; ^4^ AI: Adequate intake ^5^ [40].

## Data Availability

The data are available on request from the corresponding author.

## Supplementary Materials

The following supporting information can be downloaded at: https://www.mdpi.com/article/10.3390/molecules27227690/s1, Figure S1. The typical chromatogram of NADES extracts of licorice (NADES5) (A), glycyrrhizic acid 0.2 mg/mL (B), and pure NADES (C).
