# Supplementary material for: The Ability of Acid-Based Natural Deep Eutectic Solvents to Co-Extract Elements from the Roots of Glycyrrhiza glabra L. and Associated Health Risks
Source: Molecules. 2022 Nov 9;27(22):7690. doi: 10.3390/molecules27227690 (PMC9694035; doi:10.3390/molecules27227690)
Supplement: Supplementary file 1 [file molecules-27-07690-s001.zip › molecules-1994191-SI.pdf]

Supplementary materials

The ability of acid-based natural deep eutectic solvents for co-extraction of elements from roots of *Glycyrrhiza glabra* L and associated health risks

Alexander N. Shikov [1,*], Veronika A. Shikova [1], Anastasia O. Waley [1], Marina A. Burakova [1], Elena V. Flisyuk [1], Andrew K. Whaley [1], Inna I. Terninko [1], Irina V. Gravel [2,] and Olga N. Pozharitskaya [3]

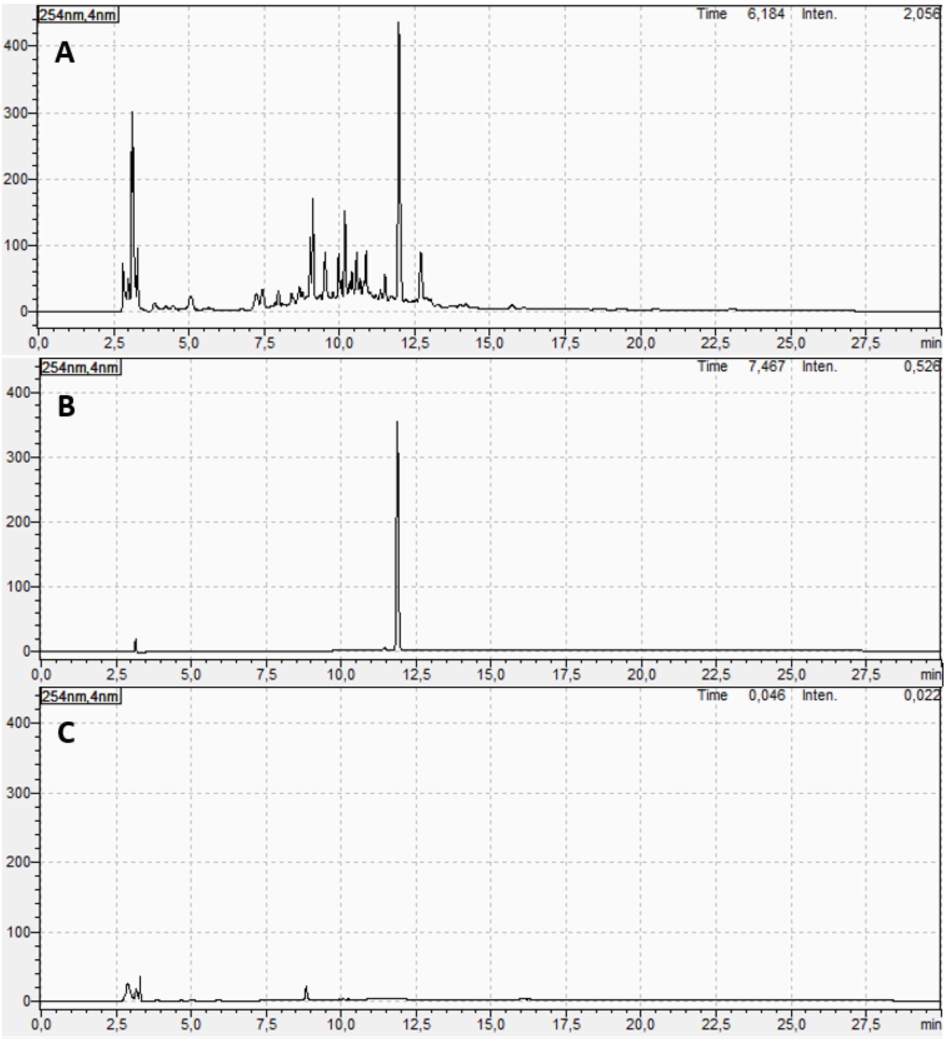

Figure S1. The typical chromatogram of NADES extract of licorice (NADES5) (**A**), glycyrrhizic acid 0.2 mg/mL (**B**), and pure NADES (**C**).
